# *Ex vivo *infection of human embryonic spinal cord neurons prior to transplantation into adult mouse cord

**DOI:** 10.1186/1471-2202-11-65

**Published:** 2010-05-29

**Authors:** Gábor Márton, Dóra Tombácz, Judit S Tóth, András Szabó, Zsolt Boldogköi, Ádám Dénes, Ákos Hornyák, Antal Nógrádi

**Affiliations:** 1 Department of Ophthalmology, Faculty of General Medicine, University of Szeged, Szeged, Hungary; 2 Department of Medical Biology, Faculty of General Medicine, University of Szeged, Szeged, Hungary; 3 Laboratory of Molecular Neuroendocrinology, Insitute of Experimental Medicine, Budapest, Hungary; 4 Department of Virology, Central Veterinary Institute, Budapest, Hungary; 5 Dept. of Pharmacology, University of Oxford, Mansfield Road, Oxford, OX1 3QT, UK

## Abstract

**Background:**

Genetically modified pseudorabies virus (Prv) proved suitable for the delivery of foreign genes to rodent embryonic neurons *ex vivo *and maintaining foreign gene expression after transplantation into spinal cord in our earlier study. The question arose of whether human embryonic neurons, which are known to be more resistant to Prv, could also be infected with a mutant Prv. Specifically, we investigated whether a mutant Prv with deleted ribonucleotide reductase and early protein 0 genes has the potential to deliver marker genes (gfp and β-gal) into human embryonic spinal cord neurons and whether the infected neurons maintain expression after transplantation into adult mouse cord.

**Results:**

The results revealed that the mutant Prv effectively infected human embryonic spinal cord neurons *ex vivo *and the grafted cells exhibited reporter gene expression for several weeks. Grafting of infected human embryonic cells into the spinal cord of immunodeficient (rnu-/rnu-) mice resulted in the infection of some of the host neurons.

**Discussion:**

These results suggest that Prv is suitable for the delivery of foreign genes into transplantable human cells. This delivery method may offer a new approach to use genetically modified cells for grafting in animal models where spinal cord neuronal loss or axon degeneration occurs.

## Background

The introduction of proteins into transplantable neurons is a possible way to study the role of these proteins in the survival or the regeneration of the grafted neurons. Several gene delivery systems have been developed with the aim of achieving this goal, including non-viral and viral vector-based methods. The viral-based systems offer greater potential as they permit efficient and cell-specific transduction.

Pseudorabies virus (Prv) possesses a complex enveloped icosahedral morphology and a large (142 kb), linear double-stranded DNA encoding over 70 genes. The virus is a promising candidate for gene delivery into the nervous system since it infects neurons with high efficiency and can acquire large exogenous DNA. In neurons, Prv either replicates and produces lytic infection, resulting in the death of the infected cell, or remains in a latent state. The replicating virus spreads through the synaptically linked neurons, and its attenuated strains, e.g. the Bartha strain [1], are widely used in neuroanatomical tracing experiments [2-5]. In the latent phase, the total viral genome is transcriptionally silent except for a restricted DNA segment which produces the latency-associated transcripts [6]. The *trans*-activator early protein 0 (*ep0*) plays a role in the reactivation of the virus from the latent state, and the neurovirulence determinant ribonucleotide reductase (*rr*) is essential for the virus replication in non-dividing cells [7-9]. Accordingly, to produce a mutant Prv that is non-replicative in embryonic neurons, both the *ep0 *and *rr *genes must be deleted. In our earlier study therefore, a mutant Prv was engineered by deleting the *ep0 *and *rr *genes (Prv-rrep0lacgfp), which made the virus unable to replicate in neurons. The Prv-rrep0lacgfp virus containing green fluorescent protein (*gfp*) and *lacz *genes proved suitable for the delivery of marker genes into rat embryonic spinal cord neurons and for the maintenance of stable expression of these genes for several weeks following transplantation [10]. Recently, mutant Prv was used to deliver troponeon into canine cardiomyocytes [11], and it also proved to be suitable for combined tract tracing and gene delivery [12].

Earlier experiments have shown that despite being a non-human pathogen, Prv successfully infects various individual human cells (hepatoma and neuroblastoma cells and embryonic spinal cord neurons) [13]. These results raised the question of whether the mutant Prv can be used to induce and maintain foreign gene expression in transplanted human embryonic neurons infected with Prv prior to grafting. The aim of the present study was to investigate the efficacy of the genetically engineered Prv in infecting human embryonic spinal cord neurons *ex vivo *and in establishing gene expression after transplantation.

## Results

### Infection of human embryonic neurons

We first investigated the toxicity of the Prv-rrep0lacgfp virus to the human embryonic spinal cord neurons after incubation times of 6 h and 12 h with the virus at a titer of 10^8 ^p.f.u. using the Trypan Blue exclusion test. Untreated human embryonic spinal cord pieces served as control. After the 6 h incubation with the Prv, 79% ± 6 (SEM) of the cells remained viable, this number decreased to 58% ± 8 (SEM) after incubation for 12 h. The proportion of viable cells in the Prv-incubated tissue pieces did not differ significantly (paired *t*-test) from that in the control tissues at any time point (p_6h _= 0.65; p_12h _= 0.4).

**Figure 1:**
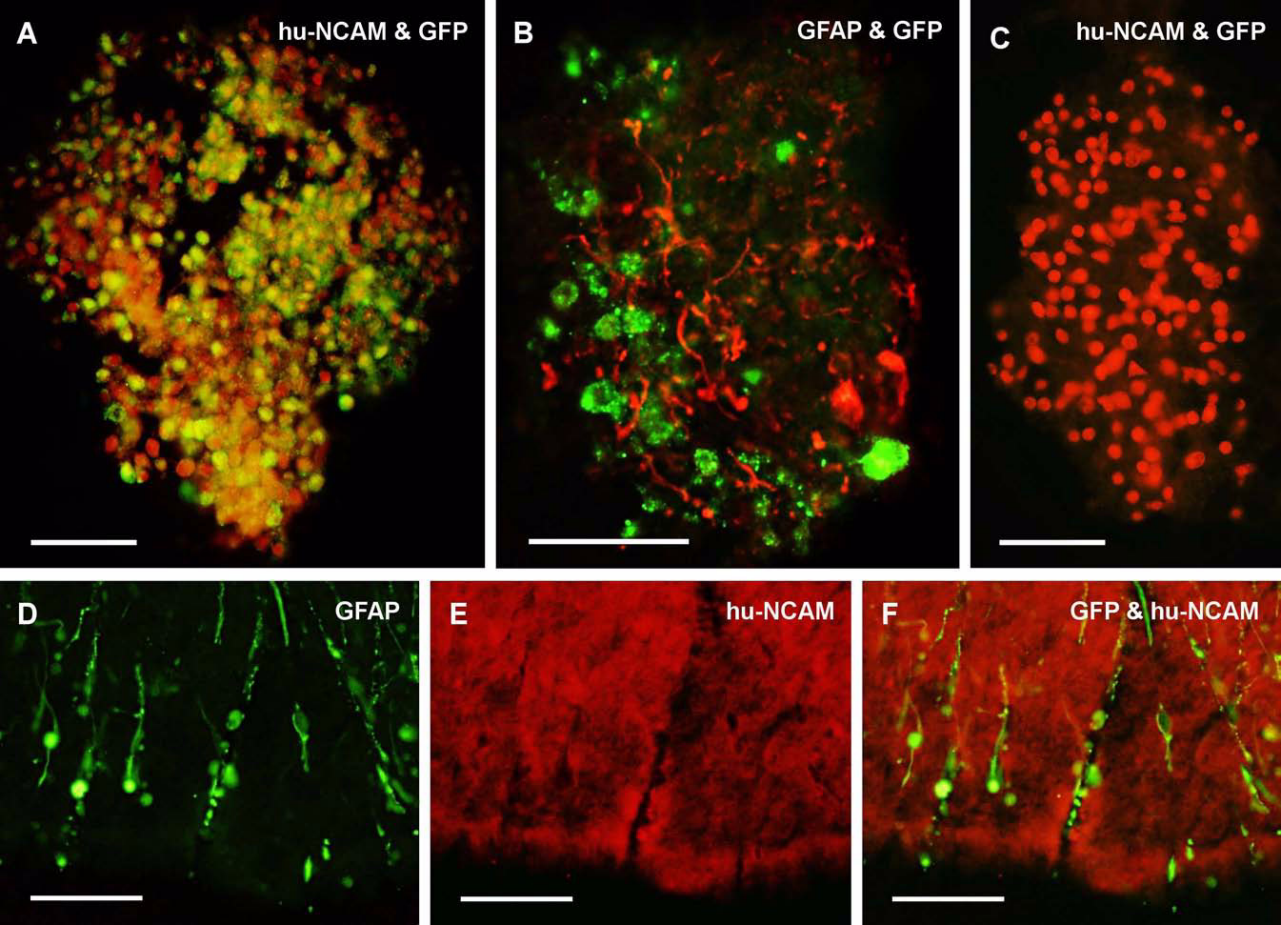
**Untreated and virus treated human embryonic spinal cord cells**. **A **and **B **shows 8.5 weeks-old spinal cord cells incubated for 12 h with the Prv. In **A**, the human cell marker hu-NCAM is shown in red and GFP is green. In **B**, the GFAP+ cells are red and GFP is green. **B **shows embryonic spinal cord cells at higher magnification. The cord is poor in cells and the two types of immunolabelling can be clearly distinguished: there is no overlap between the GFP and the GFAP labellings. Note the relatively well-developed glial cells with extensive processes. In **C**, result of the mock-infection can be seen: Embryonic cells are positive for NCAM, but contain no GFP. Scale bar in A-C: 50 μm. **D, E **and **F **show 12-weeks-old untreated human embryonic spinal cord cells processed for GFAP and hu-NCAM double labelling immunohistochemistry. In **D**, the glial cells are green, and in **E**, the human cell marker hu-NCAM is red. In **F**, the merged image of **D **and **E **is shown. It should be noted, that the two types of immunolabelling do not overlap. The glial cells shown in **E **carry a radial glia-like morphology. Scale bar: 250 μm

Next, the ratio of cells expressing foreign genes was determined in cryostat sections of the human embryonic tissues incubated with Prv-rrep0lacgfp virus at a titer of 10^8 ^p.f.u. for 6 h or 12 h, using double labelling immunohistochemistry against GFP and hu-NCAM. In previous experiments the Prv-rrep0lacgfp virus was able to infect rat embryonic neurons efficiently after incubation for 3-6 h in a solution containing 10^8 ^p.f.u./ml of Prv and the incubation resulted in an infection rate of 100% [10]. However, in our present experiments incubation for 6 h time was found not to be long enough to achieve the infection of the majority of the human embryonic tissue (not shown). After incubation for 12 h, however, the majority of the cells (85% ± 2.8 (SEM) exhibited strong β-gal or GFP labelling throughout the whole piece of the cord (Fig. 1A).

We also examined the cell specificity of the Prv infection in cryostat sections of the human tissues incubated with the Prv for 12 h by using GFAP and GFP double-labelling immunohistochemistry. The virus induced GFP expression in many cells of the embryonic tissues, but none of them was GFAP-positive (Fig. 1B). Mock-infection without virus resulted in intense hu-NCAM labelling of the embryonic spinal cord cells without GFP immunolabelling (Fig. 1C). The specificity of hu-NCAM immunolabelling was tested on human embryonic spinal cord cells: the hu-NCAM antibody did not label GFAP-positive cells in the spinal cord (Fig. 1D,E,F), and it therefore appeared evident that the Prv infected neurons only. The GFAP immunolabelled glial cells had a radial glia-like appearance in the peripheral parts of the embryonic cords (Fig. 1D and 1F), while more centrally located glial cells had an appearance of cells with more developed processes (Fig. 1B). The presence of radial glia in embryonic human cords during this early phase of neural development has already been confirmed [14].

**Figure 2:**
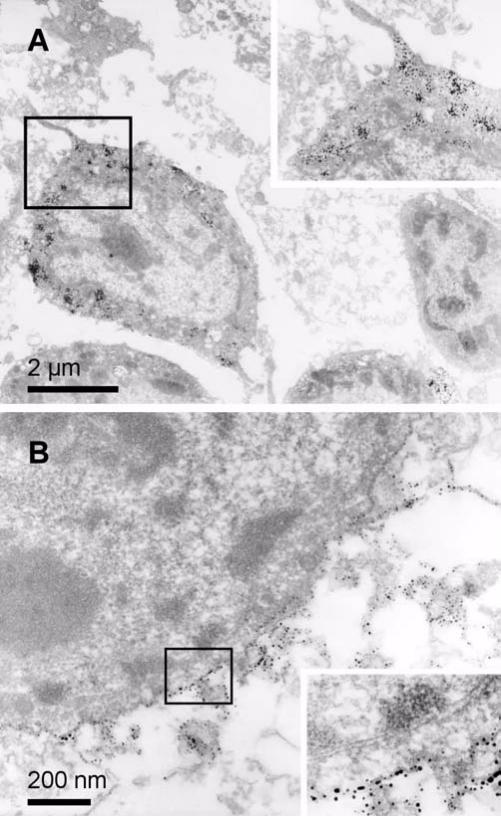
**Electron microscopic photographs of human embryonic spinal cord tissue treated with the Prv for 12 h**. In **A**, the green fluorescent protein immunohistochemistry end-product is labelled by silver granules. The boxed area at high magnification reveals the reaction product in the cytoplasm. In **B**, the virion antigens are visualized in the infected human embryonic spinal cord cells. The boxed area shows at high magnification the presence of the virion antigen on the cell membrane.

The presence of the virion antigens and the expressed GFP were monitored ultrastructurally, too. The Prv antigens were confined to the cell membranes of the infected neurons and no viral antigens or the whole virion were found in the cytoplasm of the cells. The expressed GFP was present in high amounts in the cytoplasm of the cells and was not concentrated in a given part of the cytoplasm, but was dispersed equally in the soma and in the processes of the infected human neurons. The infected human cells displayed a similar morphology to that of the untreated ones (Fig. 2.).

### Marker gene expression by grafted human cells

**Figure 3:**
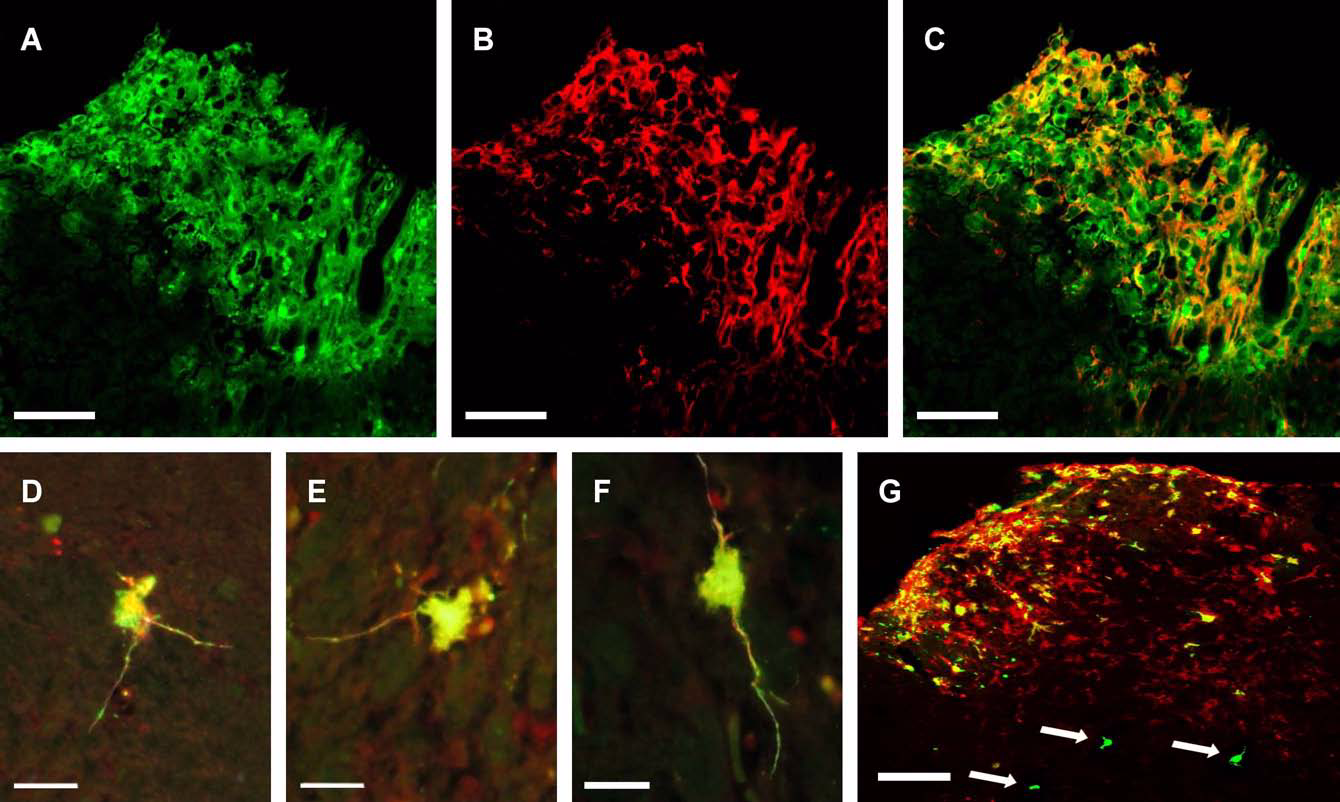
**Human spinal cord graft cells infected with Prv-rrep0lacgfp virus before transplantation**. The cells containing hu-NCAM (human cells) are red, GFP^+ ^cells are green and the cells expressing both antigens are yellow. Confocal microscopic photographs (**A, B **and **C) **illustrate the same grafted spinal cord tissue after a survival time of 1 week in the dorsal horn of the host cord. In **D, E **and **F **human neurons can be seen 3 weeks after transplantation: the human neurons express GFP and possess well-defined processes. In **G**, host cells (arrows) are shown expressing GFP near the human graft 3 weeks after transplantation. Scale bar: 40 μm

The foreign gene expression and the survival of the infected human cells grafted into the spinal cord of immunodeficient nude mice were studied next. The product of the *gfp *gene and the presence of the hu-NCAM were detected by double labelling immunohistochemistry 1 week and 3 weeks following grafting. Although the volume of the transplanted tissue was constant in each experiment, the number of the surviving human cells varied in a broad range: after 1 week, the numbers of surviving cells ranged between 410 and 2357 cells (1007.83 ± 824.8 SEM); after survival for 3 weeks 586-1358 cells remained (736.29 ± 309.63 SEM). To study the time course of foreign gene expression, we determined the number of the GFP-positive human cells in consecutive sections of the grafted cords. After survival for 7 days 79% ± 1.25 (SEM) of the human cells expressed GFP and the number decreased to 50% ± 3.12 (SEM) after survival for 3 weeks. On survival for 1 week the human cells appeared virtually undifferentiated as they had no visible processes. After survival for 3 weeks many grafted cells had well-developed, long and branching processes (Fig. 3D, E, F).

In 2 animals after survival for 7 days and in 3 animals after survival for 3 weeks GFP-expressing host neurons were found no farther than 500 μm away from the graft-host interface. The numbers of GFP-positive host cells observed in the spinal cords of the nude mice after the 1 and 3-week survival periods did not differ significantly: 26 ± 8 (SEM) cells.

## Discussion

This study has furnished evidence that a mutant neuro-attenuated Prv is able to infect *ex vivo *human embryonic spinal cord neurons and to maintain a prolonged foreign gene expression after transplantation into the spinal cord of immunodeficient mice.

In an earlier study [10] the Prv-rrep0lacgfp virus was used to introduce genes into rat embryonic spinal cord neurons prior to grafting. Similarly to those results, when applied to human tissue the gene delivery system has proved to possess the following features: a) a high efficiency of infection; b) non-cytotoxicity; c) a duration of foreign gene expression of several weeks. The above features were fulfilled by a) incubation in a high-dose viral suspension for longer incubation times than in the case of rat tissue, b) abolition of the *rr *and *ep0 *viral genes, and c) insertion of an expression cassette containing the *gfp *and *lacz *genes. The present study revealed that Prv-rrep0lacgfp is non-cytotoxic to the human embryonic cells as the Prv incubation did not increase the rate of cell death as compared with untreated tissues. This is thought to be a result of the combined inactivation of the *rr *and *ep0 *genes.

The susceptibility of human embryonic neurons to the Prv proved to be considerably lower than that of rat neurons: twice as long an incubation time was required for the infection of 85% of the human spinal cord neurons under the same conditions. The Prv was found to produce foreign gene expression in human neurons, but not in astrocytes.

These observations are in accord with our previous finding [13], that human tumour-derived cells were less susceptible than pig, mouse or rat cells to Prv. At the levels of organism and species, it is well established that humans are not susceptible to Prv at all.

Previously, Prv-rrep0lacgfp was found to display a decreasing expression pattern after the infection of rat embryonic neurons [10]. We have now demonstrated that the infection of human cells also exhibited a decreasing foreign gene expression pattern after long-term survival, but the rate of decrease was much greater than that for rat neurons. The infected rat graft cells displayed a slow decrease of marker gene expression (from 91.2% at 1 week to 86.2% by 3 weeks of survival), while 50% of the grafted human cells had ceased expressing foreign genes by 3 weeks of survival. These results indicate that human cells are more resistant to Prv infection and, possibly as a result of the resistance, the grafted cells are not able to maintain such a stable expression pattern as rodent cells. Indeed, we have shown in our earlier publications that human cells are less susceptible to Prv infection than for example porcine kidney cells for Prv infection [10,13]. The likely reason for this is the low efficiency of the virus entry into human cells, which is explained by the non-optimal virus-receptor interaction. It has been shown, that once the virus is able to enter the cell it can efficiently carry out viral functions within human cells [15]. Therefore it is thought that the duration of gene expression controlled by the LAT promoter is not related to the problem of resistance of human cells to Prv.

Data from more time points would provide a deeper insight into the pattern of expression of the infected human neurons of this nature, but unfortunately the very limited availability of human samples did not allow a long-term investigation.

Ideally, transplanted neurons should express protective proteins only in that period of their accommodation in the host nervous tissue when they establish connection with the host neurons, differentiate and reinnervate the target [16,17]. This interval following grafting in the case of spinal cord is presumed to be the first 8-10 weeks [10]. As the latency promoter of the herpes viruses produces lifelong expression of the delivered genes, we engineered a non-optimum expression cassette which contains foreign promoters (P_hCMV _and P_SV40_) between the latency promoter and the given marker gene. It appears that the expression cassette, which produced an ideal expression time-scale in rat neurons, induced only a shorter expression period in human cells. However, even short expression time periods may be very useful to increase the survival and regeneration of grafted neurons, and this application may be useful in animal models for spinal axonal and neuronal degeneration, such as amyotrophic lateral sclerosis.

The presence of some host neurons expressing the foreign gene around the human graft suggests that mutant virus can infect host neurons after transplantation. We assume that, there are secondarily infected cells among the infected host neurons, and it cannot be excluded that the Prv, even if to only a limited extent, is able to spread into the host spinal cord. The reason for the spread of the Prv is probably the weakness of the immune system of the host nude mice, i.e. the lack of T-lymphocytes. In contrast to our previous observations, where rat host spinal cords, supported by an intact and powerful immune system successfully prevented the limited spread of the mutant virus [10] we observed some infection of host neurons in the present study using immunodeficient nude mice as hosts. Hence, immunodeficiency and application of any virus treatment are incompatible processes, as indicated by the often fatal outcome of viral infections in immunodeficient patients.

## Conclusions

It has been shown that human neurons can be successfully infected *ex vivo *with a mutant Prv vector and the infected cells maintain the expression of the marker genes after transplantation into the spinal cord, although in a decreasing expression pattern. The limited spread of the Prv-rrep0lacgfp virus in the immonodeficient host animals demonstrates, that the Prv vector system is non-cytotoxic for the infected embryonic human neurons, but, similarly to other viral vectors, may be incompatible with immunodeficient host systems.

## Methods

### Cells and viruses

The generation and characterization of the neuro-attenuated pseudorabies virus (Prv-rrep0lacgfp) used in our experiments has already been described [10]. Briefly, the virus strain contains two mutations: a frame shift mutation in the small subunit of the ribonucleotide reductase gene and a large deletion in the early protein 0 gene. The gfp and lacZ reporter genes were inserted in place of the early protein 0 gene. Prv was propagated in porcine kidney (PK-15) cells. The GFP gene was placed under the control of a chimeric promoter composed of latency-associated transcript promoter (LAP) and human cytomegalovirus major IE promoter [10].

### Infection of the donor embryos

The human embryonic spinal cords derived from 8-12 weeks old embryos were obtained from abortions and used with the permission of the Human Ethical Committee of University of Szeged. The human tissues were used according to the guidelines of Helsinki Declaration of Human Rights. The lumbar enlargement of the spinal cord was dissected and the ventral portion was chopped into 1-2 mm pieces. The pieces of tissue were incubated with the virus for 6 and 12 hours at 37°C in a Sanyo CO_2 _thermostat. In each experiment 1 ml virus suspension containing 10^8 ^plaque-forming units (p.f.u./ml) of virus particles was used for the infection. Following incubation the samples were washed three times in Hanks' solution to remove excess virions. We separated 8 infected spinal cord pieces and allowed in Hanks' solution for 13 hours then processed for either Trypan Blue staining or immunohistochemistry. However, infected tissues marked for transplantation were used up immediately. Mock-infection was performed by keeping embryonic spinal cords tissues in Hanks' solution for 24 hours.

### Surgery

Adult homozygous BALB/c *rnu-/rnu- *nude mice (Charles River Laboratories, Inc., Gödöllő, Budapest, n = 15) were used as hosts for transplantation. The mice were athymic and therefore had no T-cell-based immune response. All surgical interventions were performed under deep anaesthesia achieved with a combination of Hypnorm (0.0045 ml/10 g body weight, Hypnorm, Janssen) and Diazepam (0.0075 ml/10 g body weight, from 5 mg/ml stock solution). On postnatal day 3, the left sciatic nerve of the mice was crushed to bring about motoneuron depletion in the spinal segments L4-L5. The animals were allowed to recover and used for grafting when they reached full maturity (18-20 g). After sciatic nerve crush, the animals exhibited permanent partial paralysis in the affected hindlimb. At the age of 8 weeks, the mice were anaesthetized again as described above and, after skin incision and muscle dissection, L1 laminectomy was performed corresponding to segment L4 of the spinal cord. The dura was opened to expose the cord and 0.5 μl of solid embryonic cord pieces were pressure-injected into the segment L4 via a Hamilton syringe equipped with a glass micropipette. Finally, the muscles were closed over the laminectomy site and the skin was sutured. After survival time of 7 days or 3 weeks, the animals were perfused transcardially with 4% phosphate-buffered paraformaldehyde (pH = 7.4).

### Histochemistry

The Trypan Blue exclusion test was performed on the Prv-treated human embryonic spinal cord cells after Prv incubation for 6 h or 12 h. The tissues were first trypsinated with a mixture of trypsin and EDTA, and the dissociated cells were then incubated with Trypan Blue solution (Sigma Aldrich, Budapest, Hungary) for 20 min, followed by a brief wash in physiological saline. The numbers of labelled and unlabelled cells were determined by using a Bürker chamber. The above procedure was carried out on untreated human embryonic spinal cord tissues, too. The proportions of viable cells were determined in the Prv-treated and and in the control samples.

The embryonic tissues incubated with the Prv and the grafted spinal cords were postfixed in 4% paraformaldehyde and then cryoprotected in 30% sucrose in phosphate-buffered saline overnight. Serial cryostat sections (25 μm thick) were cut in a cryostat (Leica CM1850, Bensheim, Germany) and mounted onto gelatine-coated microscopy slides.

The following antibodies were used to detect antigens: polyclonal rabbit antibody against glial fibrillary acidic protein (GFAP, 1:1000, Dakopatts, Denmark) labelling astrocytes and radial glial cells which are typical glial cells in the foetal CNS, polyclonal rabbit antibody against the whole virion (1:10.000, generous gift of Lynn Enquist, Princeton University, Princeton, NJ, USA), monoclonal mouse antibody against β-galactosidase (β-gal; 1:200, Boehringer Mannheim, Germany) and polyclonal chicken antibody against GFP (AB160901, 1:4000; Chemicon International, Temecula, CA, USA). For clarity, only the results of GFP immunohistochemistry are shown. The human neurons were detected by monoclonal mouse antibody against the human neural cell adhesion molecule (hu-NCAM, clone CD56, 1:400; IQ Products, The Netherlands). The antibody recognizes hu-NCAMs expressed on human postmitotic neurons [18]. The primary antibodies were visualized with appropriate biotinylated secondary antibodies and various fluorescent chromophores. Some immunohistochemical reactions were tyramide-amplified by using TSA-Cy3 and TSA-FITC (Perkin Elmer Life Sciences, Boston, MA, USA).

The sections were investigated in an Olympus BX50 fluorescent microscope and photographs were taken with a DP70 digital camera (Olympus Ltd., Tokyo, Japan). Some sections were investigated in a Zeiss LSM510 Meta confocal microscope, too. The labelled cells in the embryonic cords and in the grafts were identified and counted: hu-NCAM or GFP-positive cells were counted only if the cell profile was not present in the consecutive section.

The subcellular localization of virions and GFP was investigated in some Prv-infected embryonic tissues by using electron microscopic immunohistochemistry. Vibratome sections (40 μm thick) were processed for immunohistochemistry. In the immunohistochemical procedure after the ABC step, 3,3'-diaminobenzidine tetrahydrochloride (Sigma Aldrich Co. Ltd., Budapest, Hungary) was added and the oxidized coloured end-product was silver-intensified [19]. Sections were postfixed with 1% aqueous OsO_4 _and then embedded in Durcupan (Fluka, Buchs, Switzerland). Ultrathin sections (50-60 nm) were cut on an ultramicrotome (Leica Ultracut R, Bensheim, Germany) and the sections were treated with uranyl acetate and investigated in an electron microscope (JEOL JEM 1010, JEOL Ltd, Tokyo, Japan).

### Statistical analysis

Whenever needed, the paired t-test was used for the statistical analysis of data.

## Abbreviations

Prv: pseudorabies virus; GFP: green fluorescent protein; β-gal: β-galactosidase; ep0: early protein 0; rr: ribonucleotide reductase, SEM: standard error of mean; EDTA: ethylenediaminetetraacetic acid; hu-NCAM: human neural adhesion molecule; GFAP: glial fibrillary acidic protein;

## Authors' contributions

GM has been involved in performing the experiments, analysis and interpretation of data and drafting the manuscript. DT and JST took part in preparation of the Prv. AS assisted with tissue processing. ÁH took part in propagation of the virus. ÁD assisted with confocal microscopy. ZB planned the mutant Prv. AN has been involved in designing the experiments, drafting the manuscript and revising it critically. All authors read and approved the final manuscript.
